# Factors that contribute to disparities in time to acute leukemia diagnosis in young people: an in depth qualitative interview study

**DOI:** 10.1186/s12885-022-09547-8

**Published:** 2022-05-12

**Authors:** Lucky Ding, Julia E. Szymczak, Erica Evans, Emma Canepa, Ashley E. Martin, Farah Contractor, Richard Aplenc, Galen Joseph, Lena E. Winestone

**Affiliations:** 1 University of California San Francisco (UCSF) School of Medicine, San Francisco, CA USA; 2 Department of Biostatistics, Epidemiology and Informatics, Perelman School of Medicine, University of Pennsylvania, Philadelphia, PA USA; 3 Division of Neonatology, Children’s Hospital of Philadelphia, Philadelphia, PA USA; 4 PolicyLab, Children’s Hospital of Philadelphia, Philadelphia, PA USA; 5 Center for Childhood Cancer Research, Division of Oncology, Children’s Hospital of Philadelphia, Philadelphia, PA USA; 6 Center for Pediatric Clinical Effectiveness, Children’s Hospital of Philadelphia, Philadelphia, PA USA; 7 Department of Humanities and Social Sciences, UCSF, San Francisco, CA USA; 8 UCSF Helen Diller Family Comprehensive Cancer Center, CA San Francisco, USA; 9 Division of Allergy, Immunology & BMT, UCSF Benioff Children’s Hospitals, Mail Stop 0434, 550 16th St, 4th Floor, San Francisco, CA USA

**Keywords:** Disparities, Leukemia, Pediatric oncology, Qualitative research, Race/Ethnicity

## Abstract

**Background:**

Racial and ethnic disparities in outcomes for Black and Hispanic children with acute leukemia have been well documented, however little is known about the determinants of diagnostic delays in pediatric leukemia in the United States. The primary objective of this study is to identify factors contributing to delays preceding a pediatric leukemia diagnosis.

**Methods:**

This qualitative study utilized in-depth semi-structured interviews. Parents and/or patients within two years of receiving a new acute leukemia diagnosis were asked to reflect upon their family’s experiences preceding the patient’s diagnosis. Subjects were purposively sampled for maximum variation in race, ethnicity, income, and language. Interviews were analyzed using inductive theory-building and the constant comparative method to understand the process of diagnosis. Chart review was conducted to complement qualitative data.

**Results:**

Thirty-two interviews were conducted with a diverse population of English and Spanish speaking participants from two tertiary care pediatric cancer centers. Parents reported feeling frustrated when their intuition conflicted with providers’ management decisions. Many felt laboratory testing was not performed soon enough. Additional contributors to delays included misattribution of vague symptoms to more common diagnoses, difficulties in obtaining appointments, and financial disincentives to seek urgent or emergent care. Reports of difficulty obtaining timely appointments and financial concerns were disproportionately raised among low-income Black and Hispanic participants. Comparatively, parents with prior healthcare experiences felt better able to navigate the system and advocate for additional testing at symptom onset.

**Conclusions:**

While there are disease-related factors contributing to delays in diagnosis, it is important to recognize there are multiple non-disease-related factors that also contribute to delays. Evidence-based approaches to reduce outcome disparities in pediatric cancer likely need to start in the primary care setting where timeliness of diagnosis can be addressed.

**Supplementary Information:**

The online version contains supplementary material available at 10.1186/s12885-022-09547-8.

## Background

Although survival in children with acute leukemia has improved over time [1], not all children have been able to benefit equally from this progress. The racial and ethnic disparities in outcomes for Black and Hispanic children with acute leukemia have been well documented. Black patients with acute myeloid leukemia (AML) have more than a 50% increased risk of death compared to White patients with AML [2–4]. Hispanic and Black patients with acute lymphoblastic leukemia (ALL) also have an increased risk of mortality and lower event-free survival compared to non-Hispanic White patients [5–7]. Prior research has implicated lower socioeconomic status (SES) in contributing to inferior outcomes, as pediatric ALL patients residing in neighborhoods with the highest poverty have increased mortality compared with patients residing in neighborhoods with the lowest poverty [5, 8].

In solid tumors and adult cancers, later diagnosis contributes to increased morbidity due to increased cancer burden. In acute leukemia this link is less well demonstrated. However, we previously showed that Black children with acute myeloid leukemia present with higher acuity prior to chemotherapy, and that higher acuity at presentation accounts for more than 60% of the excess early mortality among Black patients [9]. The time interval from disease onset to diagnosis may be a marker of access to care [10] and can contribute to additional morbidity during the initial treatment period [11].

Previous studies have highlighted several factors that contribute to delays in pediatric cancer diagnosis, including the variety and variable timing of initial cancer symptoms, misinterpretation of non-specific cancer symptoms by parents and physicians, and tensions in the doctor-patient relationship [12–14]. More recent studies further characterize the impact of financial costs, SES [15–17], and healthcare system limitations, including insurance [18–20], on diagnostic time intervals in developing and developed nations.

However, the effect of timely access to care on pediatric acute leukemia disparities within the United States has not been studied and may differ from previous studies in light of the country’s heterogeneous population and unique healthcare system. Moreover, while two studies [21, 22] have been published investigating delays in diagnosis of childhood tumors in the US, the determinants of diagnostic delays in pediatric leukemia in the US have not been investigated. Structural barriers identified from studies in other countries that have more uniform cancer referral pathways are difficult to apply to the US due to its complex referral pathways and multiple contact points within the healthcare system. Additionally, while previous studies have examined socioeconomic and language issues in racial and ethnic differences in children’s general access to care [23], the applicability of these factors to the pre-diagnostic period in pediatric leukemia is not clear.

## Methods

### Aim

This study aimed to identify factors contributing to disparities in delays preceding a pediatric acute leukemia diagnosis by characterizing families’ experiences.

### Design, sample, and recruitment

Patients ages 0–28 years who were within two years of receiving a new diagnosis of acute leukemia at two tertiary care pediatric cancer centers were enrolled in the study. Purposive subject selection was used to maximize variation in participant race, ethnicity, socioeconomic status, and primary language. Participants in the interviews included a parent of the patient and/or a patient aged 14 years or older, who was capable of describing their path to diagnosis. Patient and/or parent/legal guardian informed consent and HIPAA Authorization were obtained prior to enrollment. All study procedures were approved by both the Children’s Hospital of Philadelphia and the University of California San Francisco Institutional Review Boards.

### Interview data collection

In-depth semi-structured interviews were conducted from June 2017 to December 2020, during which parents and/or patients were asked open-ended questions to reflect upon their family’s experience leading up to the patient’s diagnosis of acute leukemia. The interview questions served as a framework to prompt discussions of the symptoms that influenced patients to seek medical attention, the trajectory of interactions with various points of the healthcare system prior to diagnosis, and perspectives on their experiences with communication in the diagnostic process (Interview Questions available in Supplemental Table 1). Demographic and socioeconomic information were collected at the end of the interview via close-ended questions. Interviews were audio recorded and transcribed verbatim. For Spanish speaking participants, a phone interpreter was used during the interview, then the English portion of the interview was transcribed.

**Table 1:** Characteristics of study population

Patient Characteristic	n (%)
**Diagnosis**	
AML	5 (16%)
ALL	27 (84%)
**Age at Diagnosis**	
0–9 years	19 (59%)
10–19 years	10 (31%)
20–29 years	3 (9%)
**Sex**	
Male	15 (47%)
Female	17 (53%)
**Race/Ethnicity**	
Non-Hispanic White	15 (47%)
Non-Hispanic Black	3 (9%)
Hispanic	12 (38%)
Other^a^	2 (6%)
**Annual Household Income**	
Low [< $50,000]	15 (47%)
Middle [$50,000-$100,000]	6 (19%)
High [> $100,000]	9 (28%) 2 (6%)
Not reported	
**Insurance**	
Private	13 (41%)
Public	19 (59%)
**Primary Treatment Site**	
CHOP	20 (63%)
UCSF	12 (38%)
**Interviewee**	
Parent	28 (88%)
Patient	5 (16%)
**Primary Language**	
English	27 (84%)
Spanish	5 (16%)

^a^Asian, and American Indian / Alaskan Native

### Chart review

A retrospective chart review was conducted to complement the qualitative data from the interviews for each patient. Key clinical data were directly abstracted from each patient’s electronic health record and entered in a standardized form on REDCap™. Each patient’s date of diagnosis and number and type of pre-diagnostic medical encounters were collected. Additional data collected included initial symptoms/presentation, objective physical findings that prompted further laboratory workup, and subsequent workup and/or treatment following each pre-diagnosis medical encounter. These chart review data were used to discern potential pre-diagnostic clinical patterns.

### Data analysis

Interview transcripts were uploaded into Atlas.ti qualitative data analysis software and independently analyzed using inductive theory-building [24] and the constant comparative method [25, 26] by two coders (LD and EE) in a two-step progression. First, transcripts were analyzed in a process of open-coding to develop a preliminary thematic schema agreed upon by the research team. Then, interview data were independently analyzed again to assign consensus codes to passages of transcript text. Thematic saturation [25] was monitored by transcript review for recurring themes in each domain.

## Results

A total of 32 in-depth interviews were conducted with a diverse population of English and Spanish speaking participants, including 28 parents and 5 patients (27 parent-only interviews, 4 patient-only interviews, 1 joint parent and patient interview). Characteristics of the study population are summarized in Table 1. Three primary domains emerged from the narratives: structural factors, variability in leukemia presentation, and quality of interpersonal interactions. Within these domains, repeated key themes were identified to further categorize barriers and facilitators encountered by study participants along their diagnostic pathway. Themes within each domain with illustrative quotations are summarized in Table 2.

**Table 2:** Themes and illustrative quote(s)

Theme	Illustrative Quote(s)
	**Domain #1: Structural Factors**
Insurance Disincentives	*“We have a high-deductible insurance plan … so that we do tend to be a little slower to get care … I would say that if we had an insurance plan that didn’t kind of penalize us for using it, that we would probably be a little quicker.”* [high income, non-Hispanic White parent]
	*“She didn’t have any kind of medical coverage, so I asked for my bosses to help me out … they told me I was supposed to have the medical coverage from [date] onwards … I took her on the first date.”* [low income, uninsured, Spanish speaking, other Hispanic parent]
Difficulty Getting a Timely Appointment	*“I normally would have taken him to his pediatrician, but it was just the time of year where they’re so busy there – early January.”* [low income, non-Hispanic White parent]
	*“I took the decision to call the doctor, and they told me that the doctor was busy, that he had a lot of patients.”* [low income, uninsured, Spanish speaking, White Hispanic parent]
Initial Site of First Encounter	*“Urgent care just felt like they were like, eh, viral syndrome, see you later, call your pediatrician tomorrow.”* [middle income, non-Hispanic White parent]
Navigating the Healthcare System	*“She had petechiae on her legs… I know what that is, because I have low platelets myself … and so I know that that’s a sign to watch for a problem.”* [low income, non-Hispanic White parent]
	*“I just had asked my doctor to do blood work … I’m like he just doesn’t look right. I don’t know if there’s something brewing … so he ran the blood work for me … it was actually the doctor I work for.”* [middle income, non-Hispanic White parent]
	**Domain #2: Variability in Leukemia Presentation**
Timing of Symptom Onset	*“It was probably like three months that passed, and he just kept complaining about his pain in his stomach and his legs.”* [low income, non-Hispanic Black parent]
	*“I woke up one morning and I just felt really sick, like I needed to take a shower … after I took a shower I was short of breath, and I said that we’re going to the hospital.”* [low income, Hispanic Black patient]
Nonspecific Symptoms	*“He probably is running a virus. Kids run viruses all the time. It’s December, he probably has something.”* [high income, non-Hispanic parent]
Behavioral Changes	*“Basically she wasn’t acting like herself … she was very tired, and kinda dozing off in the afternoon, which is so unusual for her.”* [high income, non-Hispanic White parent]
Red Flag Symptoms Prompting Medical Attention	*“He started to develop petechiae on the upper left side of his face, near the eye, and then, towards the base of his head.”* [high income, non-Hispanic White parent]
	*“A few days later, she started having leg pain episodes again … and she couldn’t even walk with her legs. So I took her to the emergency room.”* [low income, other Hispanic, Spanish speaking parent]
Misattribution of Symptoms	*“They put it off as being allergies because they would give me medication for allergies and that wasn’t working out … it’s got to be something else. And they said well, it looks like the flu.”* [low income, American Indian/Alaskan Native parent]
	**Domain #3: Quality of Interpersonal Interactions**
Parental Intuition	*“I think a mom has a special – I don’t know, like ability to see things that people don’t see. So I knew that something was wrong with him. It just didn’t seem normal.”* [low income, non-Hispanic Black parent]
Tensions around Management Decisions and Testing	*“The only thing that I wish – and it wouldn’t have changed anything, like [he] still has leukemia, so the blood work – but it would have just been finding it a little bit sooner and getting started treatments sooner.”* [high income, non-Hispanic White parent]
	*“I think that was just the more frustrating part that I was – I knew something was wrong and I felt like it was wrong and I emphasized that something was wrong and they were just like, oh, it’s okay, everything’s okay, she’s okay.”* [low income, Hispanic White parent]
Seeking Additional Care	*“I was at urgent care for five or six hours with her. But that was already after a pediatrician’s visit the week before where I felt like there was something wrong.”* [middle income, non-Hispanic White parent]
Trust in Provider	*“They are who we trust in these situations … this is the third time I’m bringing my daughter back in a month. I just felt like would it have hurt to maybe feel her abdomen one of the first few times we came in to realize … that her liver and spleen were swollen?”* [middle income, Hispanic White parent]
	*“I just feel like something’s wrong. And she said to me without hesitation, if you think something is wrong, you go and you go right away. And she was super supportive and did not at all try to stop me. Encouraged me to go.”* [high income, non-Hispanic White parent]
Dismissal and Judgment by Providers	*“It was almost like his behavior towards treating a person that is no longer under their care … he just glanced over it like you would a piece of paper.”* [low income, non-Hispanic Black parent]
	*“[I] recall Dr. [name] yelling at me regarding [patient]’s weight loss … Dr. [name] yelled that [I] needed to cook better food for my daughter and that McDonald’s was not adequate nutrition … in reality, because [patient] had such a decreased appetite, [I] wanted her to eat anything and everything that she would in order to gain some weight back.”* [low income, Hispanic White parent]
Family Relationships and Home Environment	*“There’s a lot of aunts and uncles that have stepped up and have helped me … because we live almost three hours from [the hospital] … she’ll get the kids to school for me, pick them up.”* [middle income, non-Hispanic White parent]

### Structural factors

#### Insurance disincentives

A number of parents described insurance disincentives to seeking immediate medical attention for their child’s initial symptoms. Some cited high-deductible costs as deterrents to actually utilizing covered benefits in a more timely manner, opting to wait and see if symptoms resolved before bringing their child to the doctor. They additionally reported financial concerns that influenced the type of care that was ultimately sought, comparing the disparate costs of co-pays for urgent care and emergency visits despite both being included in insurance coverage. As one parent explained: *“Urgent Care I believe is $25 or $50 through our insurance. ER is $500 … It dissuades people. And it sounds so ridiculous because it’s your life.”* [middle income, Hispanic White parent].

In comparison, other parents who had insurance with manageable deductibles described the potential financial burden associated with the care received during both the pre-diagnostic workup and treatment period: *“If we didn’t get the HMO – we’ve got a stack of EOBs [Explanation of Benefits] here that’s $358,000 … his care maxes out at $4,000. I can’t imagine being a family in a position where you’re staring at those kind of obstacles.”* [high income, non-Hispanic White parent].

#### Difficulty getting a timely appointment

A diagnostic barrier raised by multiple study participants was difficulty getting an appointment with their primary care provider, largely attributed to lack of immediate openings in clinic schedules. *“We were managing our son’s care that week by the doctor’s hours because I was trying to see his primary and she just wasn’t working that week.”* [high income, non-Hispanic White parent].

In some cases, scheduling difficulties with the primary care provider led to visits with other providers in multi-provider practices or separate settings. Seeing different providers due to scheduling difficulties disrupted the continuity of care that otherwise helps build trusting relationships between patients and providers. As one parent described: *“There was no doctor at the university and they sent me to another doctor at another place … He did not care for my son.”* [low income, Hispanic White parent].

Some parents opted to wait for an open appointment with their primary care provider. Others opted for urgent care visits instead, which often felt counterproductive as patients were often advised to schedule follow-up with a primary care provider afterward.

#### Initial site of first encounter

Difficulty obtaining a timely appointment and differences in the burden of insurance cost-sharing between different sites influenced the site of the initial medical encounter. *“I couldn’t get him in with the pediatrician – just go ahead and take him to the urgent care.”* [low income, non-Hispanic White parent].

It is important to note that initial sites of medical encounters were not limited to primary care, urgent care, and emergency care settings. Other initial encounters associated with a possible cancer symptom included the dental office, school nurse, and other pediatric and medical specialists. One patient, without an established primary care doctor, presented first to her gynecologist, who detected anemia on initial bloodwork and subsequently referred her to a hospital: *“I didn’t go to the doctor’s before, so I haven’t even been in to see a primary ever since I was diagnosed with this.”* [low income, Hispanic White patient].

Based on chart reviews, no relationship between the location of first encounter (primary care clinic vs urgent care vs emergency department vs specialty clinic) and the total number of medical encounters prior to diagnosis was noted. However, in the emergency department setting patients more often had blood work done compared to primary care settings (63% vs 28%, respectively, as shown in Supplemental Table 2). Multiple patients with suspected leukemia due to concerning laboratory results at a local hospital were then transferred to the tertiary care center due to lack of expertise in the evaluation and treatment of pediatric leukemia; transfers were generally perceived to be timely and well-coordinated by study participants.

#### Navigating the healthcare system

Families for whom the leukemia diagnosis was their first encounter with the healthcare system for a serious illness in retrospect described feeling unable to advocate for their child during the initial diagnostic workup: *“I felt like I could have spoke out more. That I should have told them to draw blood earlier… I wasn’t thinking to be like, oh, can you all draw blood and do this, do this, and a third?”* [low income, non-Hispanic Black parent].

The complex medical concepts involved in a leukemia workup further complicated healthcare navigation for those with limited health literacy and language barriers: *“My husband went with him, but my husband didn’t understand anything they said. My husband had understood that they had told me that the x-ray came back bad, so I went the following day.”* [income unreported, other Hispanic, Spanish speaking parent].

On the other hand, parents with previous experiences with healthcare felt better positioned to navigate the medical system during their child’s diagnostic workup. Those with previous or existing medical conditions described how their personal experiences helped them recognize red flag symptoms, such as petechiae, in their child. Of note, being personally employed in the healthcare setting was reported to facilitate earlier diagnostic bloodwork, though this only applied to a small subset of participants.

### Variability in leukemia presentation

The timing of symptom onset was widely variable between participants, ranging from days to weeks. The reported symptoms of initial presentation were also variable and non-specific, including rash, fever, pallor, bruising, loss of appetite, fatigue, and pain. They were often reasonably attributed to more common, benign processes by both physicians and parents. For instance, multiple participants reported receiving a diagnosis of viral upper respiratory infection, particularly in winter months. Growing pains were another commonly reported attribution for children presenting with extremity pain: *“They just said that he was having growing pains because he was getting taller…and they kept saying the same thing.”* [low income, non-Hispanic Black parent].

Parents shared that it was often a significant change in their child’s behavior or affect that prompted them to seek medical attention, although this also presented variably and vaguely in the form of appetite changes, increased naps/sleep, and abruptly needing to be carried by parent.

### Quality of interpersonal interactions

#### Parental intuition, tensions around management decisions and testing

Tensions in parent-provider interactions were reported when parental intuition conflicted with the provider’s approach. Multiple parents expressed that their intuition alerted them to the serious nature of their child’s symptoms. They expressed frustration when medical teams did not pursue further workup for unresolved or persistent symptoms. Many respondents felt dismissed when bringing their child in for repeated visits for the same chief complaint whether they were seen by the same provider or different providers in shared group practices: *“They kept saying he has strep throat … they were giving him antibiotics for strep throat, but the fevers would not subside. And he still had the strep throat every time.”* [low income, non-Hispanic Black parent].

#### Seeking additional care, trust in provider

Seeking other additional medical care was commonly reported by parents when their impressions conflicted with management decisions made by initial providers. *“He wanted to see us back in the office the next day, but … I just felt like there was just something wrong. And so instead of waiting to see him the next day, we went into the emergency room.”* [low income, other Hispanic parent]. A breakdown in trust between parents and providers was more often described in cases involving frustration in having repeat visits for the same complaint. In some cases, this broken confidence led to changing primary care providers in the long term. *“He’s not her pediatrician. I think just not having that trust in him anymore in that if I tell you what’s going on with her are you going to believe me, are you going to look into it more … just not sure if he’ll take things serious, that was very uneasy for me.”* [low income, Hispanic White parent].

In contrast, the continuity established through previous long-standing interactions contributed to a trusting relationship between providers and parents. Parents who felt involved in the decision-making process were more likely to report trust in their provider. In a few cases, parents reported feeling encouraged by their primary care provider to obtain additional input. *“My experiences have been really positive. He is a really good doctor. He’s a doctor of the family. And he really help[ed] us with this process. … He said that maybe [patient] would need to be provided care at a different place. But he said that we needed to insist. So he made calls … and they told us okay. We’re going to see him.”* [low income, Hispanic White parent].

For some families whose primary language is not English, a combination of language discordance and health literacy barriers contributed to miscommunications about diagnostic plans: *“They told us that supposedly, they were going to perform a biopsy. The attending doctor did nothing. And supposedly given that my children can speak English and I cannot, what the doctor told them basically is that Dr. [Last Name] should instead focus on him being overweight and with the cholesterol issues.”* [low income, Hispanic White, Spanish speaking parent].

In contrast, language concordance and the use of interpreters contributed to positive care experiences with improved transparency and trust in the patient-provider relationship. When asked what made the family trust the medical team, they responded: *“Well, because they received us very pleasantly. And most of them spoke Spanish even though they were not our race.”* [income unreported, other Hispanic, Spanish speaking parent].

#### Dismissal and judgment by providers

A range of experiences involving feeling judged by providers were also reported to contribute to interpersonal tensions and a breakdown in trust between parents and providers. General sentiments of feeling dismissed or belittled by their providers were described more often by parents of low SES or minority race/ethnicity. *“I ended up getting pregnant again and he goes ‘again?’ … It made me feel like he got into my personal life and he shouldn’t have … instead of just being my doctor.”* [low income, Hispanic Black patient].

First time mothers, especially, reported feeling their concerns were dismissed by providers who attributed their worries to being a new parent. As one parent describes: *“I felt like I got pushed aside or kind of told, ‘it’s okay, don’t worry, she’s fine, everything’s okay… it happens, new babies – just get used to it, you’re a new mom, you’ve got to get used to your baby’.”* [low income, other Hispanic parent].

Such actions which appear to be rooted in implicit bias contributed to both delays in diagnostic workup and interpersonal tensions that led to a breakdown in trust between the parent/patient and provider.

#### Family relationships and home environment

Two parent households and parents who had extended family nearby, identified childcare help as a factor that allowed them to find time to bring their sick child in for medical visits. This was especially important for households with multiple children and for cases where the household was distant from medical care. Additionally, involvement of grandparents or extended family members in the regular care of the child was described as a facilitator as it provided secondary observers to recognize changes that parents had otherwise not noticed: *“My mom – she really saw it better than I … she actually had the boys a few days … she knew just playing with him that he was not okay. So she’s really the one that saw it all and was like, please, can I take him in.”* [low income, non-Hispanic White parent].

Having family members in healthcare, for parents who themselves had limited prior experiences with healthcare, was reported as a facilitator along the diagnostic pathway: *“The spots that he had on his chest – after that – I have a brother that’s a doctor. I sent him pictures over there in Peru … he’s like, you have to take him to the doctor because that is dangerous … and I was like, what is leukemia?”* [low income, Hispanic White, Spanish speaking parent].

### Pre-diagnosis medical encounters

Details of the pre-diagnostic course were generally consistent between documentation in the electronic health record (EHR) and interview respondents’ descriptions. As in the interviews, the most commonly reported presenting symptoms at the first medical encounter were documented in the EHR as fever, pain, and fatigue. Initial management typically involved symptom relief with acetaminophen or ibuprofen. While patients’ behavioral changes (such as appetite loss or increased clinginess) were more pronounced in respondent interview descriptions of symptom presentation, the medical documentation focused more on objective signs and reported physical findings. Additionally, multiple interview respondents recounted increases in severity of pain symptoms as time went on; however this progression of pain was less frequently documented in the EHR.

Medical documentation provided insight into providers’ clinical decision-making. There was good concordance between the diagnostic workup described in interviews and documented diagnostic testing or imaging. It is important to note that symptoms perceived by parents to be concerning were not necessarily judged by physicians to be red flag symptoms requiring additional diagnostic laboratory workup. As described by both study participants and in the EHR, blood tests were more often performed [1] in subsequent medical encounters than in the first medical encounter (53% vs 31%) and [2] in medical encounters involving urgent care or the emergency department than at primary care sites (Supplemental Table 2).

## Discussion

This study aimed to identify factors contributing to delays preceding a pediatric acute leukemia diagnosis by characterizing families’ experiences. This understanding of the diagnostic journey from the parent and patient perspective generated a more comprehensive picture of the roles that various factors play in hindering or facilitating a timely diagnosis. We identified three primary domains in which barriers and facilitators to a timely diagnosis can be classified: structural factors, variability in leukemia presentation, and quality of parent-provider interactions. Subthemes within each domain further illustrated the specific ways in which hurdles were encountered along the diagnostic pathway.

This study confirms the findings of previous research, specifically regarding vague versus “red flag” symptoms [13, 14]. Difference in assessments of which “red flag” symptoms should prompt additional laboratory workup by parents compared to physicians was a driving factor behind interpersonal tensions in the parent-provider relationship, as multiple parents expressed a preference for getting bloodwork done earlier. For example, a constellation of symptoms and objective findings that prompted further workup for malignancy included pallor, petechial rash, weight loss, worsening extremity pain, and gum bleeding; whereas presentations attributable to a viral upper respiratory infection, such as fever and fatigue, often did not undergo further workup initially. Laboratory draws were notably less frequent in the pediatric primary care setting compared to the urgent care or emergency setting, which was likely at least partially due to ready access to phlebotomy and laboratory facilities.

The findings from this study include a number of novel insights. First, our study highlights compounding structural and systemic barriers that affect a patient’s diagnostic experience within general pediatrics, family medicine, and emergency medicine. Difficulty obtaining a timely appointment with a primary care provider, along with the financial burden related to insurance disincentives, influenced the initial site of first medical encounter for patients. It is concerning that financial burdens and health insurance barriers serve as a deterrent causing parents to wait until symptoms reach a more critical point before seeking treatment. Families then have to deal with the logistical hurdles of either waiting for open appointments with their established primary care provider or dealing with a new and unfamiliar provider. These less than ideal diagnostic trajectories are important to address from a systems perspective [27–29] and have implications for other cancers outside of leukemia [30, 31].

Second, while there are disease-related factors contributing to delays in diagnosis, it is important to recognize that there are multiple non-disease-related factors that also contribute to delays [32]. In a diverse population with variation in race, ethnicity, language, and socioeconomic status, the risk of implicit bias and the effect of distrust may have serious implications for interactions between patients and the healthcare system [33, 34]. These findings present an opportunity to focus on the areas of distrust identified and propose potential ways to address them. Disagreements between patient/parent and providers around when diagnostic testing is indicated are expected given the often non-specific presentation of leukemia. However, the impact of providers’ assumptions and negative impressions about vulnerable patients on the care provided and the associated health outcomes must be addressed [35]. Recognizing and mitigating discrimination and implicit bias is crucial not only for creating an equitable healthcare environment for patients, but also for facilitating timely, appropriate care for patients along their diagnostic pathway [36].

Third, several facilitators were identified that should be strengthened in order to improve care for patients during and after their diagnostic journey. Multiple parents and patients expressed that transparency and open communication with the care team were elements of a positive care experience. In fact, shared decision-making improved the interpersonal relationship between the medical team and the patients’ families [37–39]. Additionally, those who had prior experiences navigating the healthcare system were better equipped to access care more efficiently, suggesting that making healthcare systems more transparent and navigable for naïve users may be important. Having language concordant advocates from within the healthcare system – in the form of providers and interpreters – was a facilitator for patients and families whose primary language was not English. Some patient families also noted the benefits of having guidance from a social work team post-diagnosis. Patient families in the primary care setting who are unfamiliar with the healthcare system could potentially benefit from the services of health navigators [40, 41].

This study has several limitations. Participants were recruited from two large tertiary children’s hospitals, which limits generalizability to patients who remain in the community setting for diagnosis and treatment. Because we interviewed respondents up to two years from their diagnosis, it is possible that important details were misremembered or forgotten. However the review of the electronic health record supported participants’ accounts of their presentation and management. Due to a technical issue, inter-rater reliability could not be quantified. However, several measures were in place to ensure concordance between the two coders. None of this study’s participants were diagnosed with leukemia following the start of the global COVID-19 pandemic and thus this study was unable to evaluate the impact of this significant shock to the healthcare system; this represents an important area for future research. Despite these limitations, the study has identified important issues that build on previous reports from other countries and specifically pertain to the unique and more heterogeneous US population.

## Conclusions

This study identified several structural factors that serve as barriers and facilitators to a timely diagnosis for pediatric patients with acute leukemia. The findings from this study have implications for other cancers and other complex medical disorders, such as autoimmune diseases [42–44], that present with similarly vague symptoms. Furthermore, evidence-based approaches to reduce outcome disparities in pediatric cancer should begin in the primary care setting, where timeliness of diagnosis can be addressed. Paradigms exist for improving scheduling in the primary care setting, however data regarding effects on fragmentation of care remain incomplete [45, 46]. Further research on the development and implementation of models of care focused on improving diagnostic care coordination between clinics and hospitals is necessary to address the needs of diverse patients.

## Supplementary Information

**Additional file 1:** **Supplemental Table 1. **Abbreviated Semi-Structured Interview Guide.** Supplemental Table 2. **Summary of Pre-Diagnostic Care.

## Data Availability

The datasets generated and/or analyzed during the current study are not publicly available due to individual privacy but are available from the corresponding author on reasonable request.

## Abbreviations

ALL: Acute Lymphoblastic Leukemia
AML: Acute Myeloid Leukemia
SES: Socioeconomic Status
PCP: Primary Care Provider
ED: Emergency Department
