# Supplementary material for: Factors that contribute to disparities in time to acute leukemia diagnosis in young people: an in depth qualitative interview study
Source: BMC Cancer. 2022 May 12;22:531. doi: 10.1186/s12885-022-09547-8 (PMC9095817; doi:10.1186/s12885-022-09547-8)
Supplement: Supplementary file 1 — Additional file 1: Supplemental Table 1. Abbreviated Semi-Structured Interview Guide. Supplemental Table 2. Summary of Pre-Diagnostic Care. [file 12885_2022_9547_MOESM1_ESM.docx]

**Supplemental Table 1. Abbreviated Semi-Structured Interview Guide**

| Details of Path to Diagnosis | When was your child diagnosed with leukemia? |
| --- | --- |
|  | Can you walk me through the period before the diagnosis? |
|  | What led you to seek treatment for your child? |
|  | How many times did you see a doctor / medical professional between your child’s first symptoms and the diagnosis of leukemia? |
| Interactions with Healthcare System Prior to Diagnosis | Did your child have a regular pediatrician? |
|  | How would you describe your relationship with your child’s pediatrician? |
|  | Were there ever issues you disagreed about? What were they? |
|  | How easy was it for you to get to the pediatrician? |
| Coping and Social Support | What was it like for you when your child was diagnosed with leukemia? What was it like for your other family members? |
|  | Who else lives with you? |
|  | Who takes care of your child / children during the day / while you are working? |

**Supplemental Table 2. Summary of Pre-Diagnostic Care**

| **Chart Finding** | **n (%)** |
| --- | --- |
| **Common Initial Illness Presentation**  Pain  Fatigue  Fever  Pallor  Petechial Rash  Bruising  Nausea / Vomiting | 15 (47%)  11 (34%)  10 (31%)  6 (19%)  5 (16%)  5 (16%)  4 (13%) |
| **Timing of First Blood Test**  Initial Medical Encounter  Subsequent Medical Encounter  Undetermined (labs done at outside hospital / site) | 10 (31%)  17 (53%)  5 (16%) |
| **Site of Blood Test Order**  Primary Care  Emergency Room or Urgent Care  Specialist | 9 (28%)  20 (63%)  3 (9%) |
| **Total Number of Pre-Diagnosis Medical Encounters**  1  2  3  4  ≥5 | 1 (3%)  12 (38%)  8 (25%)  7 (22%)  4 (13%) |
